# Enhanced Resistive Switching and Conduction Mechanisms in Silk Fibroin-Based Memristors with Ag Nanoparticles for Bio-Neuromorphic Applications

**DOI:** 10.3390/nano15070517

**Published:** 2025-03-29

**Authors:** Jongyun Choi, Seung Hun Lee, Taehun Kim, Kyungtaek Min, Sung-Nam Lee

**Affiliations:** 1 Department of IT Semiconductor Convergence Engineering, Tech University of Korea, Siheung 15073, Republic of Korea; 2 Department of Semiconductor Engineering, Tech University of Korea, Siheung 15073, Republic of Korea

**Keywords:** silk fibroin, Ag nanoparticle, memristor, resistive switching

## Abstract

This study explores the resistive switching (RS) behavior and conduction mechanisms of Ag/SF-Ag NP/Si memristors with varying Ag NP concentrations. I-V measurements confirm stable RS characteristics across 100 cycles, with consistent set and reset voltages. Increasing Ag NP concentration enhances conductive filament formation, leading to sharper switching transitions and a higher HRS/LRS ratio, w-hich increases from 43 (0 wt% Ag NP) to 4.6 × 10^4^ (10 wt% Ag NP). Log(I)-log(V) analysis reveals a conduction transition from Ohmic to Poole–Frenkel mechanisms, indicating improved charge percolation. Reliability tests show stable LRS values, while HRS exhibits greater variation at higher Ag NP concentrations. These results demonstrate that Ag NPs play a crucial role in optimizing memristor performance, improving switching characteristics, and enhancing reliability. The findings suggest that Ag/SF-Ag NP/Si memristors are promising for high-performance resistive memory and neuromorphic computing applications.

## 1. Introduction

Memristors, a class of non-volatile resistive switching (RS) devices, have gained significant attention for their potential in memory storage and neuromorphic computing [1,2]. Unlike conventional electronic components, memristors possess the unique ability to retain their resistance state even in the absence of power, making them ideal for applications in memory devices and artificial synapses [3,4,5,6,7]. Memristors function by modulating their resistance in response to applied voltage, a property that enables them to mimic biological synapses for use in neuromorphic systems [4,5]. Their capability to emulate synaptic behaviors, such as learning and memory, has positioned memristors as a key component in the development of brain-inspired computing systems [6,7]. However, challenges remain in improving their switching performance, including issues related to stability, switching speed, and energy efficiency [8,9]. Recently, bio-based materials have emerged as attractive candidates for memristor fabrication due to their biocompatibility, sustainability, and eco-friendly processing [10,11]. Among them, silk fibroin (SF), a natural biopolymer, has gained attention for its unique combination of mechanical flexibility, biodegradability, and excellent film-forming capability, making it highly suitable for flexible and bio-integrated electronics [12,13]. SF-based memristors have been explored as promising alternatives to traditional inorganic devices, offering advantages in implantable biomedical applications and bio-sensing technologies [12,13,14]. Recent advancements have demonstrated that bio-memristors utilizing silk fibroin exhibit stable resistive switching characteristics while maintaining compatibility with biological environments, highlighting their potential for bio-neuromorphic systems [15,16,17]. SF films can be easily spun, allowing for precise control over film thickness and morphology, which is crucial for the fabrication of memristor devices [12,13]. Furthermore, the ability of SF to interact with various nanoparticles (NPs) offers opportunities for enhancing its electrical properties [14,15]. However, SF alone may exhibit limitations in its switching behavior, such as low conductivity and inadequate ion mobility, which can hinder the overall performance of memristors [16,17].

To overcome these limitations, the incorporation of metal NPs, particularly silver (Ag), has gained considerable attention [14,18,19]. Silver NPs are known for their excellent electrical conductivity and the ability to facilitate the formation of conductive filaments within the memristor device [20,21,22]. Additionally, Ag NPs provide plasmonic effects that influence charge transport behavior, potentially improving memristor switching performance beyond what conventional oxide-based devices achieve [23,24,25,26]. Recent studies have demonstrated that Ag NPs, when integrated into various materials such as polymers and ceramics, can significantly enhance the RS characteristics of memristors [23,24,25]. The combination of Ag NPs with SF holds great potential for creating high-performance memristor devices that leverage the advantages of both materials. The rationale for using Ag NPs in SF-based memristors stems from their complementary properties. Ag NPs can enhance electrical conductivity and facilitate the formation of conductive filaments, which are essential for achieving reliable RS behavior [19,26,27]. Additionally, the LSPR effect from the Ag NPs may promote a more controlled charge injection process, leading to smoother transitions between high-resistance and low-resistance states [28,29]. By incorporating Ag NPs into SF, it is possible to address the inherent limitations of silk-based memristors while maintaining the biocompatibility, sustainability, and processability of silk. This approach aligns with recent developments in bio-memristors, which emphasize the impact of environmentally friendly and biologically compatible materials for next-generation memory and neuromorphic computing technologies. While bio-based materials provide sustainability and biocompatibility, silicon-based memristors offer a key advantage—regeneration capability. Certain silicon-based memristors can be electrically reset and rejuvenated, extending lifespan and improving sustainability [30]. Integrating bio-based and silicon technologies could yield hybrid memristors that combine durability, regeneration, and eco-friendliness, advancing next-generation memory devices. In this study, we investigated the fabrication and performance of Ag/SF-Ag NP/Si memristors, focusing on the effects of varying Ag NP concentrations on the RS characteristics. By contextualizing our research within the broader field of bio-memristors, this work contributes to the growing body of research on sustainable and biologically inspired electronic materials. Our findings contribute to the growing body of research on nanocomposite memristors, providing valuable insights into how the incorporation of Ag NPs can enhance the switching performance of silk-based devices.

## 2. Materials and Methods

The fabrication of an SF-based memristor begins with the extraction of SF solution. Shattered silk cocoons are boiled in a sodium carbonate (Na_2_CO_3_) solution for 60 min to remove sericin. After boiling, the SF is thoroughly rinsed with deionized (DI) water and dried. The dried fibroin is then dissolved in a lithium bromide (LiBr) solution and aged at 60 °C for 2 h. To remove excess LiBr, the solution undergoes dialysis against DI water for 48 h. Finally, centrifugation and filtering are performed to obtain a purified SF solution. To minimize these effects, we implemented a two-step cleaning process prior to memristor fabrication. First, the Si substrate underwent standard RCA wet cleaning to remove organic contaminants and metal residues. Then, a buffered oxide etch (BOE) treatment was applied to effectively remove the native silicon oxide layer, ensuring a clean Si surface before film deposition. SF solutions containing 10 nm Ag NPs at concentrations of 0, 1, 5, and 10 wt% (Sigma Aldrich, Darmstadt, Germany) are prepared. The prepared SF solution is then spin-coated onto the cleaned substrate by depositing 300 μL of the solution and spinning at 5000 RPM for 30 s. After spin-coating, the substrate is dried at room temperature for at least 10 min. Finally, an Ag top electrode is deposited onto the device using a thermal evaporator.

Characterization of the fabricated SF-based memristor is performed using various analytical techniques. Surface morphology is analyzed using optical microscopy (OM) and atomic force microscopy (AFM). Optical properties are evaluated using photoluminescence (PL) measurements with a 266 nm laser and optical reflectivity via UV-visible spectroscopy. Electrical properties of SF-based memristors are assessed using a semiconductor parameter analyzer (HP 4155A, Santa Rosa, CA, USA) and a source meter (Keithley 2614B, Beaverton, OR, USA). For the electrical characterization, the current–voltage (I-V) characteristics of the devices are measured by applying a voltage sweep from 0 to ±5 V at a constant scan rate of 0.1 V/s. The RS behavior is evaluated by performing a series of bipolar voltage sweeps, with set and reset operations monitored for each device. To analyze the RS performance, the resistance states (high-resistance state, HRS, and low-resistance state, LRS) are measured after each switching cycle. In addition, log(I)-log(V) measurements are performed to study the conduction mechanisms during the RS process.

## 3. Results and Discussion

### 3.1. Surface Morphology and Film Characteristics of SF Films Incorporating Ag NPs

**Figure 1:**
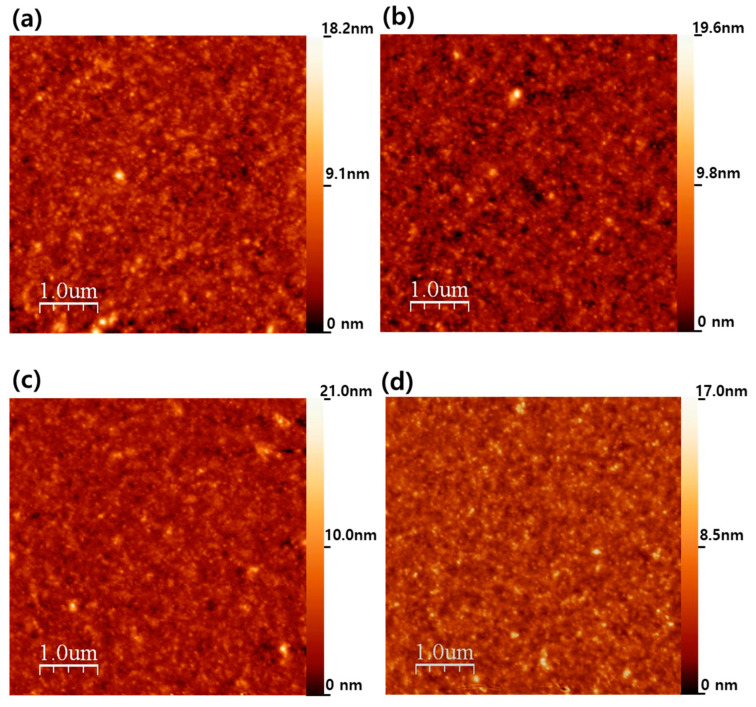
AFM images of SF films with varying Ag NP concentrations: (**a**) 0 wt%, (**b**) 1.0 wt%, (**c**) 5 wt%, and (**d**) 10 wt%. As the Ag NP concentration increases, the surface roughness slightly decreases from 1.35 nm (0 wt%) to 1.30 nm (10 wt%).

Figure 1a–d show the surface morphologies of SF films incorporating Ag NPs at concentrations of 0, 1.0, 5.0, and 10 wt%, respectively. While the overall surface morphology appears similar across all samples, the root mean square (RMS) roughness exhibits slight variations: 1.35 nm for 0 wt%, 1.33 nm for 1.0 wt%, and 1.30 nm for both 5.0 wt% and 10 wt%. This minimal variation suggests that the incorporation of Ag NPs does not significantly affect the surface roughness of SF films. The slight decrease in roughness with increasing Ag NP concentration may be due to the uniform dispersion of Ag NPs, which could reduce localized surface irregularities. Additionally, Ag NPs might influence solvent evaporation and film densification during spin coating, potentially leading to a smoother film. However, the overall roughness variation remains very small, indicating that these effects, if present, are not dominant. The stable surface roughness can be attributed to the intrinsic properties of SF, which forms a well-ordered, tightly packed structure during film formation. The strong hydrogen bonding and molecular interactions within SF [31,32] are believed to play a crucial role in preserving the smooth surface, even with the incorporation of Ag NPs. Additionally, the spin-coating process ensures uniform film formation by regulating solvent evaporation and NP distribution, preventing significant surface fluctuations. The AFM images also reveal that the SF films exhibit a relatively homogeneous and continuous morphology, with no apparent phase separation. In addition, alpha-step profiler measurements confirm that all films maintain a consistent thickness of approximately 90 nm, regardless of Ag NP concentration. This indicates that the primary film formation process is dominated by the self-assembly and drying dynamics of SF, which govern the final film thickness and morphology rather than the presence of Ag NPs.

### 3.2. Optical Properties of SF Films Embedded with Ag NPs: PL and Reflectance Spectra Analysis

**Figure 2:**
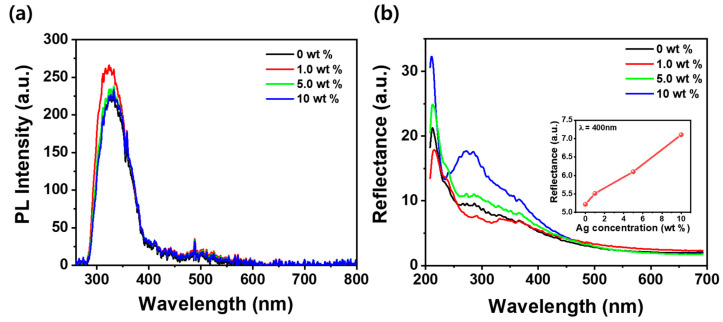
(**a**) Room-temperature photoluminescence (PL) spectra of SF films embedded with Ag NPs at concentrations ranging from 0 to 10 wt%, excited with a 266 nm laser. (**b**) Reflectance spectra of SF films with varying Ag NP concentrations. The inset shows the reflectance at 400 nm as a function of Ag NP concentration.

Figure 2a,b present the room-temperature PL spectra and reflectance spectra to analyze the optical properties of SF films embedded with Ag NPs at concentrations ranging from 0 to 10 wt%. The PL spectra show a dominant inherent peak at 330 nm when excited with a 266 nm laser, regardless of Ag NP concentration. This emission is attributed to the intrinsic fluorescence of SF, primarily originating from aromatic amino acids such as tyrosine and tryptophan [33]. Notably, at 1.0 wt% Ag NPs, the peak shifts slightly to a higher energy (around 325 nm) and exhibits the strongest intensity. This blue shift may be due to local changes in the SF matrix caused by the interaction with Ag NPs, leading to slight modifications in the electronic environment of the fluorophores. Additionally, the enhanced PL intensity at 1.0 wt% could result from an optimal coupling between the LSPR of Ag NPs and the emission process, facilitating radiative recombination. However, as the Ag NP concentration increases beyond 1.0 wt%, quenching effects [34]—likely due to non-radiative energy transfer or enhanced light scattering—may suppress further PL enhancement, thereby maintaining relatively similar intensities for higher concentrations. This behavior suggests that while LSPR interactions may play a role, the intrinsic fluorescence of SF remains the dominant factor governing the optical response. In contrast, Figure 2b demonstrates that the reflectance spectra exhibit a significant dependence on Ag NP concentration. A distinct peak appears at 290 nm, showing the weakest reflectance at 1.0 wt% Ag NPs but increasing progressively with higher Ag NP content. This trend continues across the UV region, with reflectance rising as Ag NP concentration increases. At 400 nm, the reflectance of the SF film embedded with 10 wt% Ag NPs is 36% higher than that of the pure SF film. This increase in reflectance is attributed to the LSPR effect induced by the embedded Ag NPs, which enhances light scattering and reflection at specific wavelengths. The interaction between incident light and the free electrons in Ag NPs amplifies reflectance, particularly in the UV region, where plasmonic resonance effects are more pronounced [35]. Interestingly, despite the highest PL intensity occurring at 1.0 wt% Ag NPs, the reflectance at 290 nm is the lowest at this concentration. This suggests that at 1.0 wt%, the absorption of incident UV light is maximized, likely due to enhanced coupling between SF and Ag NPs, which facilitates efficient photon absorption and subsequent radiative recombination. As Ag NP concentration increases beyond 1.0 wt%, stronger light scattering and plasmonic effects lead to higher reflectance, reducing the absorption efficiency and suppressing further PL enhancement. This inverse relationship between PL intensity and reflectance at 290 nm highlights the complex interplay between absorption, scattering, and plasmonic effects in Ag NP-incorporated SF films.

### 3.3. Electrical Characteristics of Ag/SF-Ag NP/Si Memristors: I-V Analysis Before and After Forming Process

**Figure 3:**
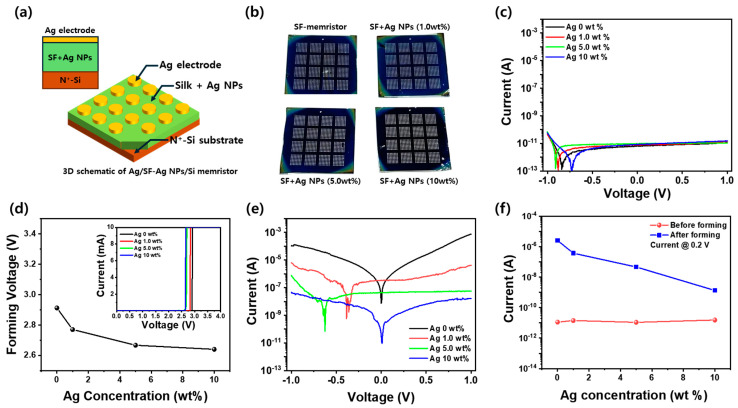
(**a**) Schematic illustrations of the cross-sectional and 3D structure of the Ag/SF-Ag NPs/Si memristor, showing the device architecture and materials layers. (**b**) Photographic image of the fabricated Ag/SF-Ag NPs/Si memristor on a 2 cm × 2 cm Si substrate. I-V characteristics of Ag/SF-Ag/Si memristor structures with varying concentrations of Ag NPs embedded in SF films: (**c**) before and (**d**) after the forming process. (**d**) Forming voltage of Ag/SF-Ag/Si memristor as a function of Ag NP concentration. (**e**) I-V curves of the Ag/SF-Ag NP/Si memristor after the forming process. (**f**) Operating current of Ag/SF-Ag/Si memristor at an applied voltage of 0.2 V before and after the forming process. Inset in (**d**) shows I-V characteristics during the forming process.

Figure 3a presents the cross-sectional and 3D schematic of the Ag/SF-Ag NPs/Si memristor, illustrating the structural configuration of the device. The diagram highlights the Ag top electrode, the SF-Ag NP active layer, and the Si substrate, showing the role of Ag NPs in facilitating conductive filament formation. Figure 3b displays a photographic image of the fabricated memristor on a 2 cm × 2 cm Si substrate, confirming the scalability and uniformity of the device fabrication process. The well-defined electrode patterns indicate that the Ag/SF-Ag NPs/Si memristor can be integrated into larger-scale memory arrays and bioelectronic applications. Figure 3c shows the current–voltage (I-V) characteristics before the formation of conductive filaments in an Ag/SF-Ag NP/Si memristor with Ag electrodes deposited on an SF film embedded with different concentrations of Ag NPs on a silicon substrate, as shown in the inset. Regardless of the Ag NP concentration, the current at an applied voltage of 0.2 V remains nearly constant, ranging from 10.8 pA to 15.3 pA. This suggests that the incorporation of Ag NPs has a minimal effect on the intrinsic electrical properties of the SF film. The stable current response indicates that the charge transport mechanism within the SF matrix is not significantly altered by the presence of Ag NPs, likely due to the strong insulating nature of SF and the uniform dispersion of Ag NPs, which do not form continuous conductive pathways at low bias conditions. The depletion voltage, which corresponds to the minimum current, exhibits a distinct pattern that becomes more negative as the Ag NP concentration increases up to 5.0 wt% and shifts to less negative values at 10 wt%. This variation in depletion voltage is influenced by the presence of Ag NPs within the SF matrix. At lower concentrations (0–5 wt%), the Ag NPs tend to act as charge traps, which impede the flow of charge carriers, resulting in a lower depletion voltage. However, at 10 wt%, the concentration of Ag NPs may lead to a slight enhancement in the formation of conductive paths, which improves charge percolation and the ability to support the electric field [36], resulting in an increase in depletion voltage. Figure 3d shows the forming voltage as a function of Ag NP concentration for the Ag/SF-Ag NP/Si memristor during the forming process, which is responsible for forming conductive channels that switch the device from high resistance to low resistance. The inset shows the I-V characteristics during the forming process of Ag/SF-Ag NP/Si memristors with different Ag NP concentrations. A sharp transition from high to low resistance is observed at each forming voltage. The forming voltage of the Ag/SF/Si memristor without Ag NPs is 2.91 V. As the Ag NP concentration in SF increases, the forming voltage decreases, reaching 2.64 V at 10 wt%. This reduction in forming voltage can be attributed to enhanced charge percolation and the increased availability of localized conductive paths with higher Ag NP concentrations. The presence of Ag NPs in the SF matrix facilitates the formation of conductive filaments, lowering the energy required for the transition to the low-resistance state and making it easier for the device to switch. Figure 3e shows the I-V characteristics of the Ag/SF-Ag NP/Si memristor after the forming process, with different Ag NP concentrations.

As shown in Figure 3f, following the forming process, the current increased significantly from the pA level to the pA~μA range, which indicates the successful formation of conductive channels and a transition to a lower resistance state. In addition, the operating current at the applied voltage of 0.2 V decreases as the Ag NP concentration increases from 0 to 10 wt%, with the current reducing from 2.56 µA to 1.35 nA. This decrease in current can be attributed to the fact that, in the case of the SF-based memristor, the high current during the forming process is due to the formation of conductive filaments induced by the Ag NPs, rather than the destruction or deformation of the SF molecular structure [26,27]. At 1.0 wt% Ag NP concentration, the embedded Ag NPs create localized conduction sites, slightly improving charge percolation while having a minimal impact on the minimum current position. As the Ag NP concentration increases to 5.0 wt%, enhanced charge trapping and stronger local electric field modulation lead to a more pronounced negative shift in the minimum current position. However, at 10 wt%, the higher density of Ag NPs promotes filament formation but also induces charge redistribution and scattering effects, partially offsetting the previous shift and resulting in a less negative minimum current position. As the Ag NP concentration increases, the conductive filaments are formed more effectively by Ag NPs, enhancing the conductive paths. However, the increase in Ag NP concentration also results in more complex current pathways, leading to current redistribution and scattering effects, which cause a decrease in overall current. This balance between charge trapping, filament formation, and field modulation explains the trend in the minimum current position observed in Figure 3e. The presence of more resistive regions due to the Ag NPs further contributes to this reduction in current.

### 3.4. RS Characteristics and Conduction Mechanisms in Ag/SF-Ag NP/Si Memristors

**Figure 4:**
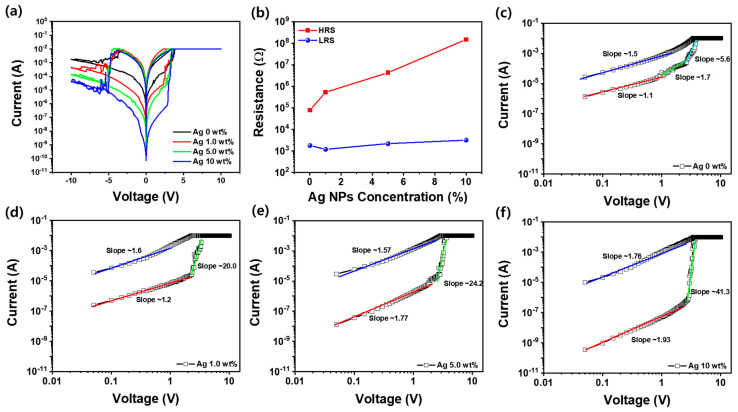
(**a**) Resistance switching characteristics of Ag/SF-Ag NP/Si memristors with varying Ag NP concentrations. (**b**) Plot of the resistance in the HRS and LRS as a function of Ag NP concentration at an applied voltage of 0.2 V. Log(I)-log(V) plots of Ag/SF-Ag NP/Si memristors with (**c**) 0 wt%, (**d**) 1.0 wt%, (**e**) 5.0 wt%, and (**f**) 10 wt% Ag NPs.

Figure 4a illustrates the RS I-V characteristics of Ag/SF-Ag NP/Si memristors fabricated with varying concentrations of Ag NPs. During the RS process, a voltage is applied from 0 V to +10 V, initiating the set process that switches the device from a high-resistance state (HRS) to a low-resistance state (LRS). Subsequently, the voltage is reduced from +10 V to 0 V, followed by a reverse voltage sweep from 0 V to −10 V to execute the reset process, which returns the device to its HRS. RS behavior is confirmed by reducing the voltage from −10 V to 0 V. These RS I-V curves demonstrate that the set voltage remains consistently around 2.5 V, regardless of the Ag NP concentration. However, a noticeable difference is observed during the set process. For Ag/SF-Ag NP/Si memristors with low concentrations of Ag NPs (≤1.0 wt%), the set voltage occurs over a relatively wide voltage range (2.0~3.5 V). In contrast, the memristor with a 10 wt% Ag NP concentration exhibits a sharp set process at a much lower current region (2.9~3.3 V), transitioning quickly from HRS to LRS. This phenomenon is attributed to the increasing concentration of Ag NPs, which act as nucleation sites for conductive filament formation. The embedded Ag NPs facilitate the reduction in Ag cations into metallic Ag under an applied field, leading to more reliable and controlled filament growth. This effect enhances charge percolation and stabilizes filament formation, reducing the stochastic nature of conductive pathways in low Ag NP concentrations. Additional Ag NPs help lower the energy barrier for filament growth, contributing to a sharper and more defined switching transition. Additionally, the reset process shows similar trends in terms of the voltage range. Memristors with lower concentrations of Ag NPs (≤1.0 wt%) exhibit reset behavior over a broader reverse voltage range. In contrast, the memristor with 10 wt% Ag NPs exhibits a very narrow voltage range for the reset process, transitioning sharply from LRS to HRS. This suggests that at higher Ag NP concentrations, the conductive filaments are more uniformly distributed and well-defined, leading to a localized and abrupt reset process. The presence of Ag NPs likely stabilizes the dissolution of filaments, reducing unwanted filamentary variations and enhancing the overall RS reliability. Figure 4b presents the values of HRS and LRS for the Ag/SF-Ag NP/Si memristor at an applied voltage of 0.2 V. The LRS increased slightly from 1.8 kΩ to 3.2 kΩ as the Ag NP concentration increased from 0 wt% to 10 wt%. However, the resistance of HRS increases significantly from 78 kΩ to 148 MΩ with increasing Ag NP concentration, showing a much larger increase compared to the relatively smaller change in the LRS. As a result, the ratio of HRS to LRS, which is a key characteristic for memristor switching, shows a remarkable increase in Ag NP-containing SF memristors. The HRS/LRS ratio increases from 43 times in the 0 wt% Ag NP memristor to 4.6 × 10^4^ in the 10 wt% Ag NP memristor, indicating a significant enhancement in the switching characteristics with higher Ag NP concentrations. Figure 4c–f show the log(I)-log(V) plots for Ag/SF-Ag NP/Si memristors with varying Ag NP concentrations: 0 wt%, 1.0 wt%, 5.0 wt%, and 10 wt%. In Figure 4c, the 0 wt% Ag NP memristor exhibits a linear region with a slope of approximately 1.1 in the low voltage region (<1.0 V) of the HRS, which is indicative of Ohmic conduction (I ∝ V). This suggests that, in the absence of Ag NPs, the conduction is primarily due to free electrons moving through the SF material without significant trapping or conductive filament formation. As the Ag NP concentration increases from 1.0 wt% to 10.0 wt%, the slope of the log(I)-log(V) plots increases from 1.2 to 1.93, as shown in Figure 4d–f. This change indicates a transition from Ohmic conduction at lower Ag NP concentrations to a Child’s law (I ∝ V^2^) mechanism at higher concentrations [6,37,38]. This transition occurs because, at higher Ag NP concentrations, the formation of conductive filaments forms a space-charge effect, limiting carrier transport and resulting in non-linear I-V characteristics. In Child’s law, the current is governed by space-charge-limited conduction (SCLS), where charge carriers injected into dielectric are influenced by the internal electric field rather than simple drift motion, leading to a quadratic dependence of current on voltage [6,38,39]. This effect enhances charge percolation and significantly influences charge transport behavior in memristors. As shown in Figure 4c, the Ag NP-free SF memristor exhibits two distinct slopes (1.7 and 5.6) near the set voltage, suggesting a transition between different conduction mechanisms. The first slope (1.7) is attributed to a transition from Ohmic conduction to Child’s law, where the current increases linearly with voltage due to the movement of free charge carriers without significant trapping or filament formation. As the voltage increases, the conduction mechanism shifts to Child’s law, which describes SCLC in the absence of a significant carrier injection barrier. The second slope (5.6) indicates a more pronounced non-linear conduction mechanism, likely due to the formation of conductive filaments or other mechanisms such as space-charge-limited conduction with trap-filled regions. In contrast, in Figure 4d–f, the Ag/SF-Ag NP/Si memristor exhibits an increasing slope in the log(I)-log(V) plot as the Ag NP concentration rises from 1.0 wt% to 10 wt%, ranging from 20 to 41.3. This trend is attributed to the enhanced formation of conductive filaments, where both the Ag electrode and Ag-doped SF layer play crucial roles. The Ag electrode serves as the primary source of Ag cations, which migrate under the applied electric field and form conductive filaments. Meanwhile, the Ag-doped SFs layer provides percolation pathways that facilitate filament nucleation and growth. The embedded Ag NPs act as localized conduction sites, lowering the energy barrier for filament formation and ensuring a more uniform switching process. During the set process, Ag cations migrate from the electrode into the SF matrix, where they reduce to metallic Ag, forming conductive bridges [26,27]. This filament-driven switching mechanism leads to a rapid increase in current, resulting in a steeper slope and a more abrupt transition from HRS to LRS as the Ag NP concentration increases. After returning to 0 V following the set process, the slope of the Ag/SF-Ag NP/Si memristor slightly increased from 1.5 to 1.76, suggesting a transition from the Ohmic conduction mechanism to the filament-dominated conduction. This change is due to the combined role of the Ag electrode and Ag-doped SF layer in filament formation. The Ag electrode continuously supplies Ag ions, while the Ag NPs in the SF layer enhance charge percolation and filament stability. As the concentration of Ag NPs increases, these filaments enhance the overall conduction behavior, leading to a higher slope, which reflects a more complex conduction mechanism compared to the initial Ohmic regime.

### 3.5. Stability and Reliability of RS Behavior in Ag/SF-Ag NP/Si Memristors

**Figure 5:**
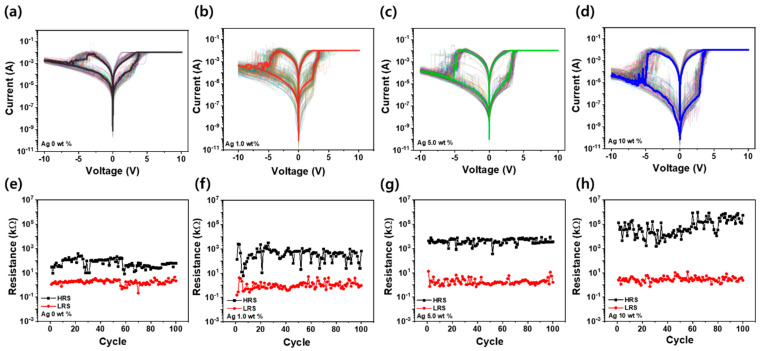
(**a**–**d**) Consecutive I-V curves of Ag/SF-Ag NP/Si memristors with Ag NP concentrations of 0 wt%, 1 wt%, 5 wt%, and 10 wt%, respectively, during 100 RS cycles. (**e**–**h**) HRS and LRS values at 0.2 V over 100 cycles for memristors with Ag NP concentrations of 0 wt%, 1 wt%, 5 wt%, and 10 wt%, respectively.

Figure 5a–d present the I-V curves of Ag/SF-Ag NP/Si memristors with Ag NP concentrations of 0 wt%, 1 wt%, 5 wt%, and 10 wt%, respectively, during 100 consecutive RS cycles. The voltage was repeatedly swept from 0 V to +10 V, +10 V to 0 V, 0 V to −10 V, and −10 V to 0 V to evaluate the stability and reliability of the RS behavior. Even after 100 cycles, the set and reset voltage ranges remained consistent, demonstrating stable and repeatable switching characteristics, similar to the trends observed in Figure 4a. Figure 5e–h show the HRS and LRS values at 0.2 V during the 100 switching cycles. Regardless of the Ag NP concentration, the LRS remained stable at a few Ω, indicating reliable conductive behavior. This stability is due to the fully formed Ag filaments in the LRS, which provide a continuous and well-defined conduction path, minimizing resistance fluctuations. However, the HRS exhibited greater variability, with increased scattering as the Ag NP concentration increased. This variability arises because the reset process does not entirely remove conductive filaments but rather partially dissolves them. At higher Ag NP concentrations, more nucleation sites exist, leading to greater randomness in filament rupture and reformation, causing fluctuations in HRS resistance. Additionally, the increased presence of Ag NPs may influence charge trapping and local electric field variations, further contributing to HRS instability. Notably, higher Ag NP concentrations led to increased HRS values, resulting in a higher HRS/LRS ratio. This suggests that Ag/SF-Ag NP/Si memristors with higher Ag NP concentrations demonstrate improved switching characteristics and data retention, enhancing the reliability of devices for memory applications.

## 4. Conclusions

In this study, we investigated the RS characteristics and conduction mechanisms of Ag/SF-Ag NP/Si memristors with varying Ag NP concentrations. The I-V measurements revealed stable and repeatable RS behavior, with set and reset voltages remaining consistent across 100 cycles. Increasing Ag NP concentration enhanced the formation of conductive filaments, leading to a sharper set transition and a more defined reset process, and reduced forming voltage. The HRS/LRS ratio significantly increased from 43 (0 wt% Ag NP) to 4.6 × 10^4^ (10 wt% Ag NP), improving switching performance and data retention. Log(I)-log(V) analysis indicated a transition from Ohmic to Poole–Frenkel conduction with increasing Ag NP concentration, suggesting improved charge percolation. Endurance analysis confirmed stable LRS values, while HRS variability increased due to the partial filament dissolution and reformation at higher Ag NP concentrations. These results demonstrate that Ag NPs play a crucial role in reducing power consumption, improving reliability, and optimizing RS behavior, making Ag/SF-Ag NP/Si memristors promising for multi-level resistive memory applications.

## Data Availability

The data presented in this study are available on request from the corresponding author. The data are not publicly available due to privacy concerns.
